# Diagnosis of Gonorrhea in the Female

**Published:** 1885-09

**Authors:** 


### Diagnosis of Gonorrhea in the Female.

Martineau, at a recent meeting of the Paris Obstetrical and Gynecological Society, stated a most important fact, by which specific can be distinguished from simple vaginitis. It depends upon this, that in the specific form of the disease the pus is always acid, while in the simple it is alkaline. It is very easy, therefore, to decide by a piece of litmus paper as to whether a woman is or is not suffering from gonorrheal inflammation. This sign will prove of value, too, in determining, when rape has been committed, whether the person committing the crime was affected with gonorrhea, for then the vulvitis would be characterized by an acid discharge, while in the simple form of the disease the discharge is alkaline.—*Weekly Med. Rev.*, etc., July 25.

